# Appraisal of Health Maintenance Organisations' Performance in the Nigerian Healthcare Service Sector

**DOI:** 10.1155/2019/6820609

**Published:** 2019-10-10

**Authors:** James O. Akinbode, Eniola A. Sokefun, Muideen O. Aremu

**Affiliations:** ^1^Department of Business Administration, Bowen University, Iwo, Osun State, Nigeria; ^2^Business Administration Department, Mountain Top University, Ibafo, Ogun State, Nigeria; ^3^Olabisi Onabanjo University, Ago-Iwoye, Ogun State, Nigeria

## Abstract

The quality of healthcare service delivery under the existing health maintenance organisations (HMOs) in Nigeria has been a major concern to enrollees who have contested the value received from their respective HMO accredited hospitals under the program. This paper appraised health maintenance organisations' performance in the Nigerian healthcare service sector capturing enrollees' experience on the issues of access, responsiveness, and quality of healthcare service choice to measure the success or failure of the program since inception. The study adopted survey design with three hundred forty enrollees of ten leading HMOs in Nigeria that operate in different parts of Lagos Metropolis. Data collected were analysed with relevant descriptive and inferential statistics while hypotheses tested were at 0.05 level of significance. Findings revealed that HMO accredited hospitals have not ensured adequate access of enrollees to healthcare services, their responsiveness to enrollees' healthcare requests have not been impressive, and quality of healthcare services to enrollees have also not been excellent. Based on the findings, the study recommends that HMOs and government should improve on monitoring the quality of healthcare service delivery at their accredited hospitals and concluded that the performance of the HMOs in the area of healthcare service delivery is not world class when it comes to access, responsiveness, and quality of service delivery.

## 1. Introduction

Good health remains one of the basic needs of individual irrespective of status in the society. This is the reason why government as a major stakeholder in any society considers the issue of health as critical and fundamental. To achieve this, government in different countries has different programs and policies to deliver qualitative healthcare system to her citizenry. Healthcare is the totality of various activities carried out to maintain optimal functioning of the individual, treatment of pathology, and the promotion of health of individuals with or without pathology [1, 2]. In spite of the importance of a healthy living to society, accessing good-quality healthcare services has been incredibly poor in developing countries [3, 4]. For Nigerians, accessing good-quality healthcare services at affordable rate has been difficult due to decay in public health institutions and expensive charges in privately owned hospitals [3, 5, 6].

One of the healthcare intervention programs in Nigeria was the introduction of National Health Insurance Scheme (NHIS) backed with Act 35 of 1999 under which Health Maintenance Organisation (HMO) was set out to address observed short fall in the quality of healthcare service delivery. The scheme is jointly financed by employer and employee who is an enrollee. The HMO model was borrowed from developed countries and treated as limited liability companies. In Nigeria, HMOs are licensed by the National Health Insurance Scheme (NHIS) to facilitate the provision of healthcare benefits to contributors.

Recent development revealed that enrollees have concerns about the value derived from the HMO programs. This observation seems true with the Nigeria low ranking in global healthcare index which covered 1990–2015 involving 195 countries placed Nigeria 187^th^ in healthcare service delivery. A strong indication of this was obvious when twenty-three HMOs in the country by National Health Insurance Scheme governing council on 5 April 2018 seemed to make the fears of enrollees about the performance of HMOs true. It was against this perception that this study was found germane to appraise the level of performance of this arrangement after over a decade of operations. Specifically, the study sought after the following:

- To examine the extent to which enrollees' healthcare service accessibility is made possible by the HMOs
- To ascertain the effect of responsiveness to enrollees' healthcare-related request on HMO performance
- To investigate the extent to which healthcare service quality enjoyed by enrollees contribute to HMO performance

## 2. Statement of Hypotheses

  H_1_: there is no significant relationship between enrollees' healthcare service accessibility and HMO performance
  H_2_: responsiveness to enrollees' healthcare-related request has no significant effect on HMO performance
  H_3_: there is no significant relationship between healthcare service quality and HMO performance

## 3. Literature Review

Healthcare system can be described as service providing entities consisting of components or subdivisions oriented towards improvement of the health status of the populace [1]. The Nigeria healthcare system is organised into primary, secondary, and tertiary healthcare levels. Grassroot government handles the primary healthcare, and state government takes care of secondary healthcare, while state and federal governments handle tertiary healthcare and also provide policy direction and regulation. Regrettably, Nigeria with an estimated total of 23,640 health facilities operated via a three-tiered governance structure, and the country is ranked 187^th^ by the World Health Organization (WHO) among 195 member states on health issues [3]. This development implied dysfunctional health system of the country because the healthcare service system lacked state of healthcare infrastructure, short of medical professionals, and other issues required in meeting international healthcare standards [7]. However, different intervention programs have been floated by government, and all seems not working.

One of such programs was the HMO arrangement which was meant to facilitate easy and qualitative healthcare services to Nigerians. The World Health Organization (WHO) within this context defines health service delivery as the way inputs are combined to allow the delivery of a series of interventions or health actions [8]. As noted in the World Health Report 2000, “the service provision function (of the health system) is the most familiar; the entire health system is often identified with just service delivery.” The report states that service provision or service delivery is the chief function the health system needs to perform [8]. The question is whether HMO has achieved the objective of WHO in Nigeria.

Health Maintenance Organisations (HMOs) are limited liability companies licensed by the National Health Insurance Scheme (NHIS) to facilitate the provision of healthcare benefits to contributors under the Formal Sector Social Health Insurance Program (FSHIP) to interface between eligible contributors, including voluntary contributors and the healthcare providers. Existing arrangement makes HMOs to either be for-profit or not-for-profit private health insurance companies, or public entities [9]. Irrespective of the motive of any of the arrangement, delivery of sound healthcare services remains ultimate. It is therefore imperative to appraise how the HMOs have performed, and this study measured it from the enrollees' perspective.

Performance in relation to individual and organisation has been extensively discussed in the literature. This has made the concept “performance” to have attracted numerous measures depending on the perspective of whosoever is defining it [10, 11]. This review concentrates on three of such measures, namely, accessibility, responsiveness, and quality [12–14].

### 3.1. Accessibility

Access to healthcare services remains one of the disturbing global issues in spite of the need and persistent call for it. Access to health care was centered on affordability, however, in this context; it refers to the function of “location of accredited hospital” to enrollees. Access also implied physical access to the service provider address, including the ease of finding ways on service provider location [15]. This is because accessing good-quality healthcare services has been problematic in developing countries like Nigeria. According to Eboh et al. [3], access to quality health care delivery in Nigerian remains a high-profile challenge and this apparently calls for research on why it has remained so in spite of the efforts of different stakeholders.

### 3.2. Responsiveness

Responsiveness represents the willingness to provide prompt services to customers [16]. According to Nicole and Gouke [17], responsiveness is the degree to which legitimate expectations of the population with respect to nonclinical aspects of health care or public health services were actually met. Precisely, it is considered as how well the hospitals under HMO arrangements meet the legitimate expectation of enrollees in covered areas of their health plans [12]. In this study, responsiveness is measured with the opinion of enrollees on healthcare service delivery experience with reference to care.

### 3.3. Quality

Extant studies have shown that quality is a vague concept, and that is why it has attracted different definitions as many as the number of authorities that have attempted to conceptualise it [18, 19]. Besides this, time and events have also made the concept dynamic [20]. According to Crosby [21], quality is conformance to requirements. In this regard, it is meeting and exceeding expectations. In context, the definition of Øvretveit [22] on quality is found applicable to this review. According to him, quality care entails the provision of care that exceeds patient expectations and achieves the highest possible clinical outcomes. Therefore, quality healthcare is doing things rightly and ensuring continuous improvements, the best possible clinical outcome that would satisfy patients [14]. From the view of patients who are enrollees, quality is obtaining adequate treatment and cure from any ailment whenever they visit hospital.

Extant studies have shown some related links in this area of review [12, 23]. For instance, the study of Akinwale et al. [23] among artisans in Lagos State which examined their reactions to the National Health Insurance Scheme confirmed limited accessibility to subsidised health services in Nigeria while that of Mohammed et al. [12] which assessed the responsiveness of healthcare services within a health insurance scheme in Nigeria from the users' perspectives revealed that communication, dignity, and quality of facilities were highly responsiveness domains with lower contentment for prompt attention, autonomy, and their confidentiality. Similarly, the study of Osuchukwu et al. [24] found that enrollees are satisfied with services offered by HMOs. This is an impression that the HMOs are doing very well in monitoring the services rendered by the accredited HMOs.

**Figure 1:**
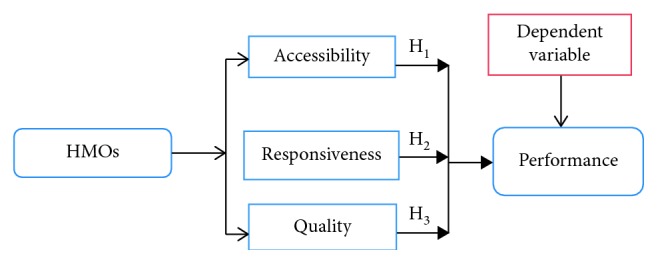
Hypothesised model of HMOs and healthcare service performance.

For the purpose of the study, the hypothesised model in Figure 1 shows that the measures of HMO performance (accessibility, responsiveness, and quality) have been used to appraise Nigerian healthcare service delivery. The three proxies were used to develop hypotheses with which HMO performance was appraised.

## 4. Methodology

Survey design was adopted for the study to collect primary data required. Enrollees of HMOs were the human subject of the study, and questionnaire was the instrument used to collect data from the enrollees' of the ten HMOs targeted in Lagos State at different areas of the State to investigate the performance of these HMOs on healthcare services delivery. The enrollees that participated were registered with one of the following HMOs: Total Health Trust Limited, Hygeia HMO Limited, Healthcare International Limited, Clearline International Limited, Cygnet Health Limited, PHB Healthcare Limited, Diamond Shield Health Services Limited, Medexia Limited, Capex Medicare Limited, and Health Wyse Global Services Limited. Simple random sampling technique was used to pick the enrollees. Data analysis was based on three hundred forty completed copies of questionnaire returned within four weeks of field survey (May 21–June 15, 2018). Data collected were analysed with inferential statistics (Pearson product-moment correlation (PPMC) and regression).

## 5. Results

### 5.1. Respondents Demographic Data

The distribution of respondents personal data is shown in Table 1.

 
Table 2 shows the result of first hypothesis tested. The results of the analysis established the extent of relationship between enrollees' healthcare service accessibility and HMO performance. The correlation *r* = 0.214 implies that there is a weak positive relationship between enrollees' healthcare service accessibility and HMO performance. The null hypothesis (H_0_) was rejected, and the alternate hypothesis was (H_1_) accepted. This implies that there is a relationship between enrollees' healthcare service accessibility and HMO performance.
  H_2_: responsiveness to enrollees' healthcare-related request has no significant effect on HMO performance.

  The model in Table 3 shows how much of HMO responsiveness to enrollees' healthcare-related request can be used to determine the performance. In this case, *R* square is 0.314, which can be expressed in percentage as 31.4%.
  H_3_: there is no significant relationship between healthcare service quality and HMO performance.

 
Table 4 reveals the extent to which healthcare service quality is related to HMO performance. The correlation *r* = 0.522 implies that there is a positive relationship between healthcare service quality which is related to HMO performance. However, the strength of the relationship is moderate, as obtained from the table (*r* = 0.522, *p* < 0.05, *n* = 340). Coefficient of determination (COD) was calculated to know the degree of healthcare service quality contribution to HMO performance assessment. Coefficient of determination (COD) = *r*^2^ × 100%, and the result equals 27%. The null hypothesis (H_0_) was rejected, while the alternate hypothesis (H_1_) was accepted.

## 6. Test of Hypotheses

  H_1_: there is no significant relationship between enrollees' healthcare service accessibility and HMO performance.

## 7. Discussion of Findings

Outcomes of this study have shown that HMO performance in the Nigerian healthcare service delivery sector is not of world class range. On the demographic issues of respondents that participated in the study, female respondents patronise HMO plans than male. This may be due to their involvement in health matters and that of their wards than husbands. Similarly, all the possible age groups were represented in the survey and signify the involvement of all. It was also deduced that Lagos state which was the domain of survey to the knowledge of researchers has not been studied aside that of Akinwale et al. [23], which was centered on artisan only while the current study involved a wider human subject. Other existing related studies have been conducted in other climes. Furthermore, the state might have been adequately covered for the study considering the selection process adopted, while various categories of enrolment plans would have been covered with the cheapest plan recording the highest number of enrollees.

The first hypothesis tested revealed a weak positive relationship between enrollees' healthcare service accessibility and HMO performance. It appears that the HMOs have not really made healthcare services through the accredited hospitals accessible to enrollees' as desired. This implied that majority of the enrollees' might not have had easy access to accredited hospitals of their HMOs. This finding corroborates the study of Akinwale et al. [23] which discovered limited accessibility of subsidised health services in Nigeria among artisans which ordinarily should not be after years of the introduction of HMO in the country. It is, however, regrettable that the trend remains and questions the performance of HMOs in Nigeria. Again, the study of Eboh et al. [3] which discovered that access to quality healthcare delivery in Nigerian remains a challenge is thus reaffirmed and echoed the inability of HMO arrangement to address the challenge healthcare delivery in the country.

Also, the study established in its second hypothesis tested on HMO responsiveness to enrollees' healthcare-related request is about 31.4%. This percentage appears insignificant and unimpressive considering the importance attached to health issues and mandate of HMOs. Therefore, HMO performance seems not on a good standing after 10 years of operations in Nigeria. This outcome further justified the results of Mohammed et al. [12] which rated prompt attention of enrollees very lower in their study on the assessment of responsiveness of healthcare services in Nigeria. This brings to bear the fore old order practices of keeping patients waiting without prompt attention and providing the needed prompt support that is care-free attitude of nurses as it were to provide healthcare services desired or that should be provided to the patients in the Nigerian hospitals. On the contrary, this finding contradicts the finding of Osuchukwu et al. [24] which evaluated the impact of National Health Insurance Scheme on healthcare consumers in Calabar Metropolis, Southern Nigeria. The authors found out that enrollees are satisfied with services offered by HMOs because for enrollees to have claimed to be satisfied with services offered, it might have implied that there is high responsiveness from the accredited hospitals of the HMOs. However, other factors such as location “Calabar metropolis,” NHIS leadership in the state, and class of enrollees investigated among others influenced this outcome and made this outcome different from that of Lagos state which is the commercial nerve center of the country with high patients and comparable indices of hospital responsiveness.

Finally, the third hypothesis on the relationship between healthcare service quality and HMO performance attributed 27% contribution of healthcare service quality to the rating of HMO performance. This might imply that healthcare service quality contributes to HMO performance that is because the totality of determinants of HMO performance measure is not curtained to have been explored under literature review. However, the contribution is relatively low, and the healthcare service quality can be assumed unimpressive. It is strongly believed that this can be improved upon because the strength of healthcare services is in the level of quality services rendered to patients. This opinion was upheld in the study of Leebov and Ersoz [14] which premised quality healthcare on things rightly, ensuring continuous improvements, best possible clinical outcome that would satisfy patients. Therefore, patients with ailments and their relatives desire a timely and effective medical treatment.

## 8. Recommendations

NHIS, HMOs, and government should improve on monitoring the quality of healthcare service delivery in Nigeria. Specifically, the following recommendations are made:

- HMOs should enlist more standard hospitals to bring healthcare facilities closer to enrollees
- HMOs should strengthen their monitoring teams to accredited hospitals to ensure excellent responsiveness to enrollees' and handle their complaints about accredited hospitals swiftly
- Quality of healthcare service delivery in all accredited hospitals of HMOs as a matter of urgency must be reviewed for improved quality healthcare service

## 9. Conclusion

The performance of selected Nigerian HMOs in the area of healthcare service delivery to enrollees is not world class rated when it comes to accessibility, responsiveness, and quality which sum up to the state of service delivery in the health sector. Based on the findings, the introduction of HMOs has not really improved Nigerian healthcare delivery system as obvious in the country global health ranking status. Enrollees have expressed their dissatisfaction in the scheme, and this no doubt calls for urgent intervention.

## Figures and Tables

**Table 1:** Distribution of respondents personal data.

Filters		Frequency	Percent	Valid percent	Cumulative percent
Valid	Male	124	36.5	36.5	36.5
	Female	216	63.5	63.5	100.0
	Total	340	100.0	100.0	
Valid	18–27 years	22	6.5	6.5	6.5
	28–37 years	89	26.2	26.2	32.7
	38–47 years	128	37.6	37.6	70.3
	48–57 years	42	12.4	12.4	82.7
	58 years above	59	17.3	17.3	100.0
	Total	340	100.0	100.0	
Valid	Lagos West	115	33.8	33.8	33.8
	Lagos East	108	31.8	31.8	65.6
	Lagos Central	117	34.4	34.4	100.0
	Total	340	100.0	100.0	
Valid	Plan A	144	42.4	42.4	42.4
	Plan B	112	32.9	32.9	75.3
	Plan C	49	14.4	14.4	89.7
	Plan D	23	6.8	6.8	96.5
	Plan E	12	3.5	3.5	100.0
	Total	340	100.0	100.0	

Source: Field Survey, 2018.

**Table 2:** Relationship between enrollees' healthcare service accessibility and HMO performance.

		Enrollees' healthcare service accessibility	HMO performance
Enrollees' healthcare service accessibility	Pearson's correlation	1	0.214^*∗∗*^
	Sig. (two-tailed)		0.000
	*N*	340	340
HMO performance	Pearson's correlation	0.214^*∗∗*^	1
	Sig. (2-tailed)	0.000	
	*N*	340	340

^*∗∗*^Correlation is significant at the 0.01 level (2-tailed). Source: Computed Data (2018).

**Table 3:** Effect of responsiveness to enrollees' healthcare-related request on HMO performance.

Model summary				
Model	*R*	*R* square	Adjusted *R* square	Std. error of the estimate
1	0.627^a^	0.314	0.097	1.1463

Predictors: (constant) responsiveness. Source: Computed Data (2018).

**Table 4:** The relationship between healthcare service quality and HMO performance.

		Healthcare service quality	HMO performance
Healthcare service quality	Pearson's correlation	1	0.522^*∗∗*^
	Sig. (2-tailed)		0.000
	*N*	340	340
HMO performance	Pearson's correlation	0.522^*∗∗*^	1
	Sig. (2-tailed)	0.000	
	*N*	340	340

^*∗∗*^Correlation is significant at the 0.01 level (2-tailed). Source: Computed Data (2018).

## Data Availability

The descriptive and inferential data used to support the findings of this study are included within the article.
